# Biosorption of Cadmium by Non-Toxic Extracellular Polymeric Substances (EPS) Synthesized by Bacteria from Marine Intertidal Biofilms

**DOI:** 10.3390/ijerph15020314

**Published:** 2018-02-11

**Authors:** Juan Carlos Camacho-Chab, María del Refugio Castañeda-Chávez, Manuel Jesús Chan-Bacab, Ruth Noemí Aguila-Ramírez, Itzel Galaviz-Villa, Pascual Bartolo-Pérez, Fabiola Lango-Reynoso, Carolina Tabasco-Novelo, Christine Gaylarde, Benjamín Otto Ortega-Morales

**Affiliations:** 1 Tecnológico Nacional de México/Instituto Tecnológico de Boca del Río, Laboratorio de Investigación en Recursos Acuáticos LIRA, Kilómetro 12, Carretera Veracruz-Córdoba, Boca del Río 94290, Veracruz, Mexico; juanccam@uacam.mx (J.C.C.-C.); castanedaitboca@yahoo.com.mx (M.d.R.C.-C.); itzelgalaviz.itboca@gmail.com (I.G.-V.); fabiolalango@yahoo.com.mx (F.L.-R.); 2 Departamento de Microbiología Ambiental y Biotecnología DEMAB, Universidad Autónoma de Campeche, Colonia Buenavista, San Francisco de Campeche 24039, Campeche, Mexico; manjchan@uacam.mx; 3 Instituto Politécnico Nacional, Centro Interdisciplinario de Ciencias Marinas (CICIMAR), Laboratorio de Microbiología y Biología Molecular. La Paz 23096, Baja California Sur, Mexico; raguilar@ipn.mx; 4 Departamento de Física Aplicada, Centro de Investigación y de Estudios Avanzados (CINVESTAV-Mérida) Instituto Politécnico Nacional, Mérida 97310, Yucatán, Mexico; pascualbartolo@gmail.com (P.B.-P.); carolinatabasco@gmail.com (C.T.-N.); 5 Department of Microbiology and Plant Biology, Oklahoma University, 770 Van Vleet Oval, Norman, OK 73019, USA; cgaylarde@gmail.com

**Keywords:** aquatic environments, bioremediation, biosorption, cadmium, extracellular polymeric substances

## Abstract

Cadmium is a major heavy metal found in polluted aquatic environments, mainly derived from industrial production processes. We evaluated the biosorption of solubilized Cd^2+^ using the extracellular polymeric substances (EPS) produced by *Bacillus* sp. MC3B-22 and *Microbacterium* sp. MC3B-10 (Microbactan); these bacteria were originally isolated from intertidal biofilms off the coast of Campeche, Mexico. EPS were incubated with different concentrations of cadmium in ultrapure water. Residual Cd^2+^ concentrations were determined by Inductive Coupled Plasma-Optic Emission Spectrometry and the maximum sorption capacity (Qmax) was calculated according to the Langmuir model. EPS were characterized by X-ray photoelectron spectroscopy (XPS) before and after sorption. The Qmax of Cd^2+^ was 97 mg g^−1^ for Microbactan and 141 mg g^−1^ for MC3B-22 EPS, these adsorption levels being significantly higher than previously reported for other microbial EPS. In addition, XPS analysis revealed changes in structure of EPS after biosorption and showed that amino functional groups contributed to the binding of Cd^2+^, unlike other studies that show the carbohydrate fraction is responsible for this activity. This work expands the current view of bacterial species capable of synthesizing EPS with biosorbent potential for cadmium and provides evidence that different chemical moieties, other than carbohydrates, participate in this process.

## 1. Introduction

Cadmium (Cd^2+^) is a transition metal present in the aquatic environment through geochemical processes and, increasingly, through anthropogenic industrial activity; it can accumulate in aquatic life along the food chain. The metal is found as a constitutive ingredient in pigments, plastics stabilizers, solar panels, batteries and corrosion-resistant steel plating [1], and thus is an important industrial material, but also a toxic waste product. Cadmium is one of the heavy metals with major toxic effects on human health and the environment [2,3,4] and has been reported in many countries to be the cause of serious health risks in contaminated areas e.g., in China [5].

Several methods have been employed for the removal and recovery of heavy metals, dyes and radionuclides from polluted environments, including electrolysis, flotation, ion-exchange, membrane process precipitation, reverse osmosis and ultrafiltration [6,7,8]. These “traditional” methods are not cost effective, efficient, or ecofriendly. Recently, considerable attention has been paid to the possibility of using bioremediation for the removal of heavy metals, including cadmium, from contaminated soil and water [9,10]. Biosorption is an applied biotechnology for the removal or recovery of organic and inorganic pollutants in solution using biological material, which includes agricultural and industrial waste biomass, living or dead microorganisms (and their extracellular products), seaweed, plant and animal material [8,11]. There are many publications suggesting the use of living bacterial cells for the removal and recovery of heavy metals from solution [12,13,14], but this procedure has the obvious drawbacks of requiring metal-resistant, non-pathogenic organisms and of providing suitable growth conditions.

Because of their chemical and physical properties, bacterial extracellular polymeric substances (EPS) have been effectively and successfully used in the removal and recovery of several heavy metals [15,16,17]. EPS are mainly dominated by biomolecules such as carbohydrates, proteins, nucleic acids and lipids, with a lower proportion of other monomeric constituents [18,19] and their biosorption capacity is attributed to ionizable functional groups such as amino, carboxyl, hydroxyl, phosphate and sulfate, present mainly in polysaccharides and proteins [16,20].

Microbial EPS have been studied from both terrestrial and aquatic environments. Specific biotopes such as intertidal flats represent understudied habitats for bioprospection of EPS-producing bacteria [21]. In an earlier investigation, bacterial strains *Microbacterium* sp. MC3B-10 and *Bacillus* sp. MC3B-22 were isolated from intertidal biofilms and were selected for their EPS production. The EPS were characterized and polysaccharides and proteins identified as major components. Their surfactant activity was also evaluated [22]. In the case of EPS synthesized by *Microbacterium* sp. MC3B10, calcium was seen bound, suggesting a potential biosorption potential. This EPS was characterized as a non-toxic glycolipoprotein, which was named Microbactan [23]. On the other hand, EPS produced by *Bacillus* sp. MC3B-22 an anionic assay proved positive, also suggesting this potential. This work aimed at determining the potential of EPS produced by intertidal *Bacillus* sp. MC3B-22 and *Microbacterium* sp. MC3B-10 to adsorb solubilized Cd^2+^ as a prior step to towards developing a biological strategy for bioremediation of cadmium-polluted waters.

## 2. Materials and Methods

### 2.1. Bacterial Strains

*Bacillus* sp. MC3B-22 and *Microbacterium* sp. MC3B-22 were originally isolated from pristine rocky intertidal shores in the state of Campeche, southern Gulf of Mexico, Mexico. They are stored in the culture collection of the Department of Environmental Microbiology and Biotechnology, DEMAB, Universidad Autónoma de Campeche. *Bacillus* sp. MC3B-22 was identified to species level by DNA sequencing, as described in Section *Bacillus* sp. MC3B-22 Identification.

#### *Bacillus* sp. MC3B-22 Identification

*Bacillus* sp. MC3B-22 was identified by partial sequencing of 16S rRNA gene fragments amplified using the polymerase chain reaction (PCR). Total DNA was obtained with a commercial kit (UltraClean^®^ Microbial DNA Isolation Kit MO BIO, (QIAGEN INC, Illinois, IL, USA) according to the manufacturer’s instructions modified according to [24,25]. In brief, the pure strain was placed in eppendorf tubes with 500 μL of TE buffer pH 8.0, 30 μL of 10% sodium dodecyl sulfate (SDS) and 5 mL of proteinase K (20 mg mL^−1^). They were vortexed and incubated for one hour at 37 °C. After this time, 100 μL of 5 M NaCl and 80 μL of a solution of cetyltrimethyl ammonium bromide in sodium chloride (CTAB/NaCl) added, mixed by inversion and incubated for 10 min at 65 °C. Subsequently phenol: chloroform: isoamyl alcohol (25:24:1) was added, vortexed and centrifuged at 14,000 rpm for 10 min at 24 °C; the supernatant was recovered and transferred to 1.5 mL sterile tubes. A second extraction with an equal volume of chloroform: isoamyl alcohol (24:1) was made by inversion mixing. The tubes were centrifuged at 14,000 rpm for 5 min at 4 °C. The aqueous phase was recovered and DNA precipitation was carried out by adding 0.6 volumes of precooled isopropanol at −20 °C. The tubes were incubated at −20°C overnight. After incubation, the tubes were centrifuged at 14,000 rpm for 20 min at 4 °C. The resulting DNA was washed with 70% ethanol precooled at −20 °C and centrifuged again; this process was repeated. DNA was allowed to air dry and resuspended in 100 μL of TE pH 8.0 plus 1 μL of RNAse (1 μg μL^−1^). The tubes were incubated in a water bath at 37 °C for 1 h. A second extraction with phenol: chloroform: isoamyl alcohol (25:24:1) was performed to remove RNase following the above-mentioned procedure.

Oligonucleotide primers (forward primer 27f 5′-GAGTTTGATCCTGGCTCA-3′ and reverse primer 1385R 5′-CGGTGTGTRCAAGGCCC-3′) were used to amplify the 16S rRNA gene. The amplification reaction contained 100 ng of DNA, 0.25 μM of each oligonucleotide, 5 μL of 10X PCR buffer, 2 μL of MgSO_4_, 10 mM dNTPs, 1.5 U of Taq polymerase and sterile distilled water to a final volume of 50 μL. The reactions were amplified in a BIO-RAD MJMini^®^ thermocycler (version 1.1 Ci, Bio-Rad Laboratories, Inc., Hercules, CA, USA). A single PCR cycle consisted of initial denaturation at 95 °C for 2 min and 30 cycles of 95 °C for 60 s, annealing at 55 °C for 60 s, 72 °C for 70 s, and extension at 72 °C for 10 min. The PCR amplicons were purified and DNA was sequenced by MACROGEN, Korea. The sequence was analyzed and edited using the CodonCode Aligner program. The Genbank database was searched using the BLAST program (version 2.2.25, NIH National Institute of Health, Bethesda, MD, USA) at the National Center for Biotechnology Information website (http://www.ncbi.nlm.nih.gov) and sequencing data was analyzed comparing the sequence of nearest relatives found by BLAST searching.

### 2.2. EPS Production

Both EPS were produced by overnight batch shake flask fermentation as previously reported Ortega-Morales et al. [21]. EPS were extracted from the fermented broths as described by Camacho-Chab et al. [22]. The EPS were lyophilized and kept in the dark before experiments. Both EPS have been analyzed and polysaccharides predominate in their composition, with glucose as main neutral sugar; uronic acids (galacturonic acid and glucuronic acid) and hexosamines (*N*-acetyl glucosamine) are also present and protein content is 38% for Microbactan and 9% for MC3B-22 EPS [21].

### 2.3. Cadmium Biosorption Experiments

50 mg dry EPS were placed in a flask with 50 mL Cd^2+^ solution at initial concentrations in the range of 10–100 (10-unit increments) mg L^−1^. A stock solution of cadmium (100 mg L^−1^) was prepared by dissolving Cd(COOCH_3_)_2_ (Sigma-Aldrich, St. Louis, MO, USA) in ultrapure water. Flasks were prepared in triplicate and gently shaken (50 rpm) for 24 h at 28 °C and pH 7 [26,27]. After 24 h, solutions were ultrafiltered using Pellicon^®^ Tangential Flow filtration cassettes (Merk Millipore, Darmstadt, German) [26]. Permeates (residual cadmium concentrations) were acidified with HNO_3_ (0.5 N) and cadmium concentration was determined by Inductive Coupled Plasma-Optic Emission Spectrometry (ICP-OES, Perkin Elmer Optima 8300 DV, Wellesley, MA, USA) at a wavelength of 226.502 nm. Retentates were lyophilized prior to further analysis by XPS. Suitable controls were included during biosorption experiments to rule out cadmium adsorption to glass flasks; these were triplicate flasks containing EPS (at varying levels) plus cadmium and flask containing only cadmium.

The amount of cadmium adsorbed, *q* per unit mass of EPS (mg g^−1^) was determined using the following equation [28]:

(1) $$q=\frac{\left(Ci-Ceq\right)\times V}{m}$$

where *Ci* is the initial metal concentration and *Ceq* the equilibrium metal concentration in solution (mg L^−1^) of volume *V* (mL) and m is the mass of EPS (mg).

#### 2.3.1. Equilibrium Biosorption Isotherm

The Langmuir adsorption isotherm model was employed to describe the Cd^2+^ uptake by Microbactan and MC3B-22 EPS [29]. This adsorption isotherm is given by the following equation:

(2) $$q=\frac{Q_{max}\times K\;\times \left[Me\right]eq}{1+K\;\times \left[Me\right]eq}$$

where *Qmax* (mg g^−1^) represents the maximum sorption capacity, *K* is the Langmuir equilibrium constant (L mg^−1^), and [*Me*]*eq* (mg L^−1^) is the equilibrium concentration of the metal in the solution.

#### 2.3.2. X-ray Photoelectron Spectroscopy (XPS)

The composition and oxidation state of surface elements of both EPS were determined by XPS before and after sorption. Analyses were carried with an X-ray photoelectron spectrometer K-Alpha (Thermo Scientific, Waltham, MA, USA) with a hemispheric analyser at 5 × 10^−9^ mbar; the sample was excited with monochromatic radiation of Al Kα (1486.68 eV) at 12 kV and 40 W. The general XPS spectra was taken with a pass energy of 100 eV and step size of 1 eV and the individual XPS spectra were taken with a pass energy of 50 eV and step size of 0.1 eV.

### 2.4. Toxicity Test

*Artemia* sp. (brine shrimp) is an important food for aquaculture, since it can be produced in gnotobiotic conditions and can serve as a vector, transferring probiotics to larvae, thus being a suitable model in the study of how probiotics and pathogenic microorganisms affect [30,31] one of the most important live feeds for commercial production of fish and shellfish [30]. *Artemia* nauplii have also been used to evaluate the toxicity of contaminants in aquatic environments [31]. The toxicity of the EPS was evaluated by the brine shrimp assay. Brine shrimp eggs (Equinetos Productos^®^, TAM, Ciudad de México, Mexico) were hatched in artificial seawater, prepared with 38 g/L of sea salt (Coralife^®^, Franklin, WI, USA) added with 6 mg/L of dry yeast, under light and continuous aeration at 27 °C. MC3B-22 EPS and Microbactan, together with standard commercial polymers alginate and xanthan gum (for comparison), were all dissolved separately with seawater at different concentrations (1000, 500, 100, 50 and 10 µg/mL). Copper sulfate pentahydrate was used as a positive control. After incubation of the test for 24 h, dead and live nauplii were counted with the aid of a stereoscope and data were analyzed by the Probit program to determine the lethal concentration of 50% (LC50) of each compound [22].

## 3. Results and Discussion

### 3.1. Molecular Identification of Bacillus sp. MC3B-22

**Figure 1:**
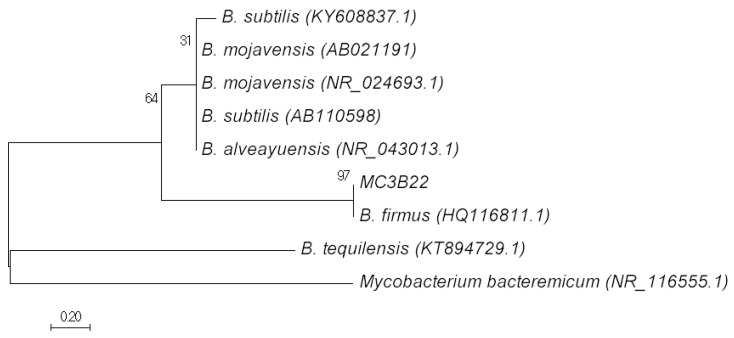
Evolutionary taxonomic relationships.

The phylogenetic analysis based on the 16S rDNA sequences indicated strain MC3B-22 to be of the genus *Bacillus* (Figure 1). The strain was closely related to *Bacillus firmus*, with a similarity value of 99% (GeneBank Accesion number HQ116811.1). The EPS produced by *B. firmus* has previously been reported to remove copper, lead, and zinc from aqueous solutions [32], but this is the first time, to our knowledge, that it has been shown to be effective for Cd sorption.

The evolutionary history of the current isolate was inferred using the Neighbor-Joining method [33]. Figure 1 shows the sum of the length of the branch that was 5.236499, as well as the percentage of trees replicated with associated taxa grouped in the test (1000 replicates) [34]. Evolutionary distances were used to infer the phylogenetic tree and were calculated using the Maximum Composite Likelihood Method [35]. Evolutionary analyses were performed on MEGA7 [36], using *Mycobacterium bacteremicum* as the outside group.

### 3.2. Cadmium Biosorption Isotherms of Microbactan and B. firmus EPS

**Figure 2:**
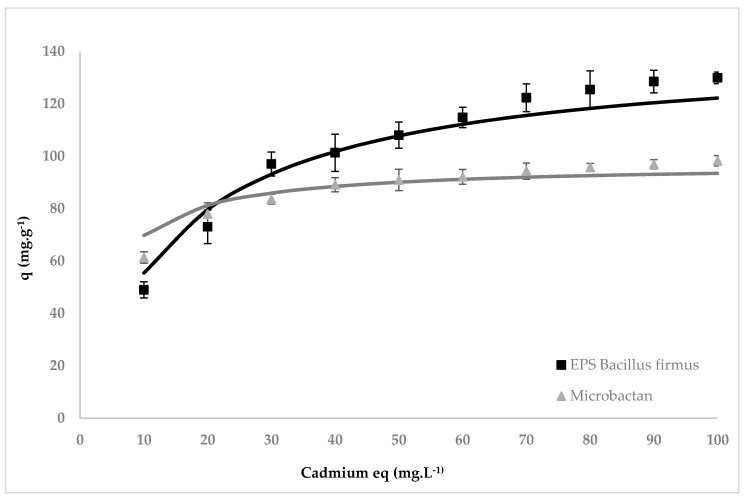
Equilibrium sorption isotherm of cadmium (10–100 mg L^−1^) for Microbactan and MC3B-22 (*B. firmus*) EPS (1 g L^−1^) at pH 7 and 28 °C. Error bars represent ± standard deviation of triplicate samples.

The sorption isotherms of cadmium for both EPS are shown in Figure 2.

The simulation of the Langmuir isotherm model showed that both EPS have the ability to sorb to cadmium and that this metal accumulation is a saturated physicochemical process. Table 1 gives the biosorption characteristics of the two types of EPS. The maximum amount of cadmium biosorption on *B. firmus* EPS (*Qmax* = 141.1 mg g^−1^) was greater than on Microbactan (*Qmax* = 97.1 mg g^−1^). Both these figures are considerably greater than most of those reported in the literature for live or dead microbial biomass [12,37]. Ahmed et al. [37] compared the *Qmax* values for various bacteria, fungi and algae. All were lower than our EPS values, apart from those for the immobilized *Bacillus subtilis* used by Ahmed et al. [37], which was calculated from a model to be 251.91 mg g^−1^, and that for the dead biomass of the endophytic fungus *Microsphaeropsis* (247.5 mg g^−1^) [38].

### 3.3. XPS Analysis before and after Biosorption

These analyses allowed us to determine the interactions occurring in EPS on Cd sorption.

#### 3.3.1. *B. firmus* EPS

**Figure 3:**
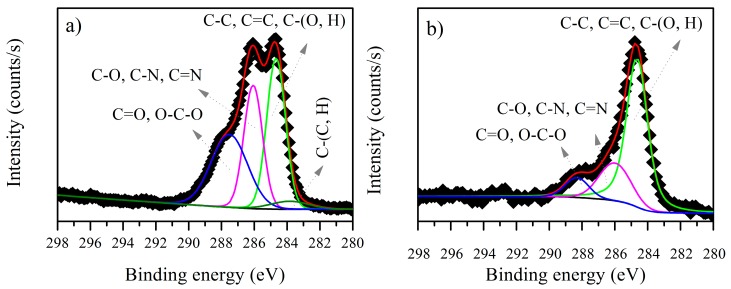
C1s X-ray photoelectron spectroscopy (XPS) spectra for *B. firmus* extracellular polymeric substances (EPS) (**a**) before and (**b**) after biosorption of cadmium.

The XPS C1s spectra for the *B. firmus* EPS before and after biosorption of cadmium are shown in Figure 3. Before biosorption (Figure 3a), the C1s spectra displays four environments for C atoms, which could correspond to C–H, C–C or C=C, C–O and C=O. The 286.05 eV peak, pertaining to C–O or C–N, was attributed to alcohol, amine or amide. The peak at 287.50 eV, pertaining to C=O or O–C–O, could be attributed to carboxylate or carbonyl, which confirms the presence of polysaccharides in *B. firmus* EPS. On the other hand, the C1s spectra after biosorption of cadmium (Figure 3b) displayed only three environments for C atoms, which correspond to C–C or C=C, C–O and C=O. A change in the binding energy of these components is observed (Table 2). The contribution of C–O and C=O decreases after biosorption, while the C–C or C=C contribution remains almost the same. Thus, the change in the C atoms could indicate that the biosorption process is modifying the *B. firmus* EPS, or that the carbonyl groups are taking part in the metal ion coordination [28,39,40]. XPS analysis has previously shown that the EPS are subject to a change in their chemical structure or conformation during the biosorption process [16].

**Figure 4:**
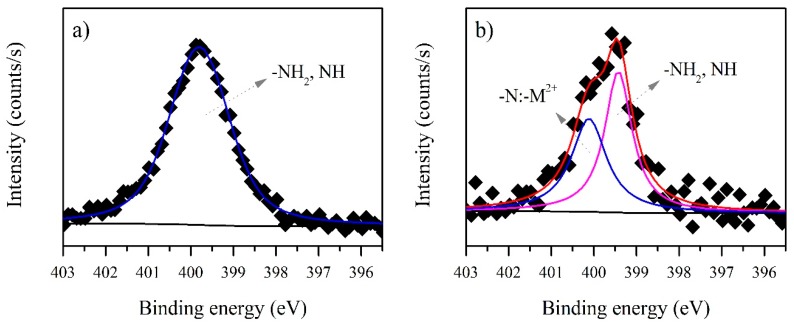
N1s XPS spectra for *B. firmus* EPS (**a**) before and (**b**) after biosorption of cadmium. M^2+^ = Metal ion.

Figure 4 shows the N1s spectra. The presence of a peak characteristic for nitrogen is seen at 399.78 eV before biosorption (Figure 4a) and this was attributed to NH_2_ or NH of the proteins. After biosorption, a new peak appeared in the N1s spectrum (Figure 4b) at the higher binding energy of 400.11 eV (Table 2) and this could be assigned to the formation of an –N:–M^2+^ complex, a lone pair of electrons in the N atom being donated to cadmium [41]. This complexation between metal ions and the NH_2_ or NH has been reported previously in chitosan [41,42,43], but not for microbial EPS. The N1s spectra thus confirm the biosorption of cadmium by *B. firmus* EPS.

#### 3.3.2. Microbactan

**Figure 5:**
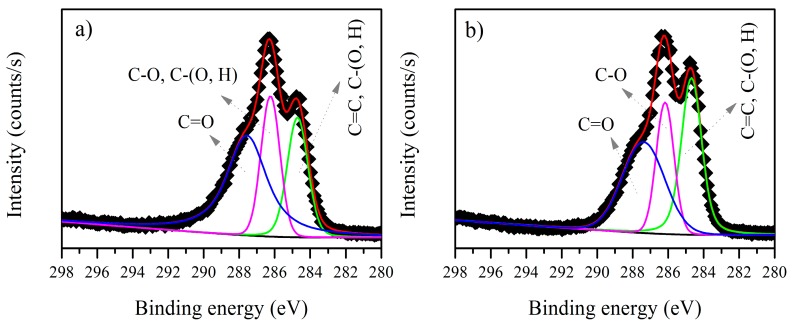
C1s XPS spectra for Microbactan (**a**) before and (**b**) after biosorption of cadmium.

**Figure 6:**
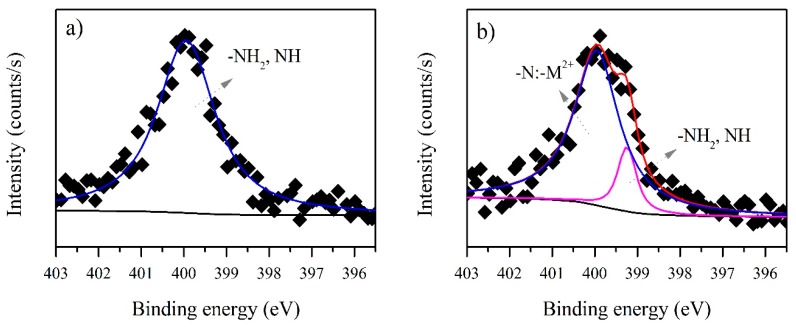
N1s XPS spectra for Microbactan (**a**) before and (**b**) after biosorption of cadmium. M^2+^ = Metal ion.

The C1s XPS spectra for Microbactan before and after biosorption of cadmium are shown in Figure 5. Both C1s XPS spectra (Figure 5a,b) show three C atom environments which were assigned to C–C or C=C, C–O and C=O. However, for three C atoms a change of peak intensity ratios is seen. A possible explanation for this is that Cd^2+^ in NH_2_–metal complexes may have a weak interaction with adjacent hydroxyl groups (286.22 eV) (Table 3) because of its relatively larger radius [39]. Alternatively, the decrease in intensity of peak ratio 287.55 eV (Table 3) after the biosorption process could be caused by an ion exchange [42]. The N1s XPS spectrum before biosorption (Figure 6a) shows the characteristic peak for nitrogen at 399.93 eV, attributed to NH_2_ or NH (protein fraction). After biosorption, a new peak appears, indicating that metal complexation has occurred with NH_2_ or NH. The N1s spectra after cadmium biosorption (Figure 6b), displays a shift in the binding energy; since a higher band corresponds to a more oxidized state, the peak at 399.95 was assigned to the –NH_2_ metal complex (Table 3). The results show that nitrogen-containing groups in both types of EPS play an important role in cadmium biosorption.

### 3.4. Toxicity of Microbactan and B. firmus EPS

The toxicity bioassay showed that neither biopolymer was toxic to *A. salina nauplii* at concentrations of 1000 µg mL^−1^ (Table 4). There are no suggestions in the literature that bacterial EPS can be toxic, although this property is rarely measured specifically. These results confirm what is generally assumed by microbiologists and the many uses of EPS in water treatment [20] suggest that this is, indeed, the case. Alginate and xanthan gum were used for comparison, as commercial biopolymers that have been reported to have biosorption abilities. The results show that the new EPS extracts are equally suitable for use in the environment.

## 4. Conclusions

This study determined the biosorption capacity of cadmium by the biopolymers Microbactan and MC3B-22, both synthetized by marine bacteria *Microbacterium* sp. MC3B-10 and *Bacillus* sp. MC3B-22 (identified by DNA sequencing with 99% similarity to *B. firmus*). The maximum sorption capacity of Cd^2+^ was 97 mg g^−1^ for Microbactan and 141 mg g^−1^ for *B. firmus* EPS, both at pH 7 and 28 °C. XPS analysis revealed changes in structure or conformation of both EPS after biosorption and indicated that NH2 or NH functional groups contributed to binding cadmium. In addition, Microbactan and *B. firmus* EPS were non-toxic to *A. salina nauplii*, which is an aquatic model organism widely used in aquaculture activities. Our results showed that *B. firmus* EPS and Microbactan are very promising for remediation of cadmium in aqueous solutions, with *B. firmus* perhaps being preferred because of its higher maximum sorption capacity. EPS does not depend for its metal-absorbing activity on microbial metabolic processes and is thus preferable to living biomass. Further studies of these EPS in remediation of heavy metals in water for aqueous systems, including optimization of their production, will be performed in the future.

## Figures and Tables

**Table 1:** Langmuir parameters (*Qmax* and K) and the correlation coefficient (*R*^2^) for the biosorption of cadmium by Microbactan and *B. firmus* EPS.

EPS	*Q_max_* (mg g^−1^)	*K* (L mg^−1^)	*R*^2^
Microbactan	97.12	0.254	0.954
*B. firmus*	141.10	0.064	0.990

**Table 2:** Binding energy and area for the C and N atoms in *B. firmus* EPS, before and after biosorption of cadmium.

Element	Peak (eV) before Biosorption	Assignment	Peak (eV) after Biosorption	Area before Biosorption	Area after Biosorption
C1s	283.82	C–(C, H)	-	1984.1	-
C1s	284.65	C–C	284.63	18,133	18,644
		C=C			
		C–(O, H)			
C1s	286.05	C–O	285.98	14,729	6053.3
		C–N			
		C=N			
C1s	287.50	C=O	288.28	16,978	2351.4
		O–C–O			
N1s	399.78	NH_2_	399.42	5593.1	970.79
		NH			
Interaction	-	–N:–Cd^2+^	400.11	-	809.53

**Table 3:** Binding energy and area for the C and N atoms in Microbactan, before and after biosorption of cadmium.

Element	Peak (eV) before Biosorption	Assignment	Peak (eV) after Biosorption	Area before Biosorption	Area after Biosorption
C1s	284.69	C=C	284.67	12,005	21,890
		C–(O, H)			
C1s	286.22	C–O	286.16	12,383	14,376
		C–(O, H)			
C1s	287.55	C=O	287.37	24,212	23,695
N1s	399.93	NH_2_	399.23	2263.6	361.49
		NH			
Interaction	-	–N:–Cd^2+^	399.95	-	2175.5

**Table 4:** Anticrustacean activity of biopolymers against *Artemia salina* nauplii.

Biopolymers	IC_50_ µg mL^−1^
Microbactan	>1000
*B. firmus* EPS	>1000
Alginate	>1000
Xanthan gum	>1000
CuSO_4_·5H_2_O (Positive control)	9.89 ± 4.69
